# Cold water ingestion ameliorates increase in core temperature and discomfort during simulated motor racing in a hot environment: a randomized trial

**DOI:** 10.3389/fspor.2025.1514963

**Published:** 2025-02-18

**Authors:** Stephen Richard Bird, Olga Troynikov, Chris Watson, Marc Cohen, Simon Sostaric

**Affiliations:** ^1^ School of Health Sciences, Swinburne University of Technology, Melbourne, VIC, Australia; ^2^ School of Engineering, Royal Melbourne Institute of Technology University, Melbourne, VIC, Australia; ^3^ Extreme Wellness Institute, Melbourne, VIC, Australia; ^4^ Melbourne Sports and Allied Health Clinic, Essendon, VIC, Australia

**Keywords:** thermoregulation, heat, motor racing, cold fluid, exercise, core temperature

## Abstract

**Introduction:**

Formula One and other motor car racing drivers race for prolonged periods in hot conditions wearing protective apparel that impairs heat loss. They are thus at risk of a significantly elevated core temperature. The aims of this study were to determine whether the voluntary ingestion of cold fluid aided thermoregulation more effectively than the voluntary ingestion of ambient temperature fluid in a simulated motor racing environment.

**Methods:**

Eight male participants commenced two 120-min simulated motor racing trials in an environmental chamber (40°C, 50% humidity). During one trial they were provided with 1 L of ambient temperature water (AWT), whilst in the other trial the water temperature was ∼5°C (CWT). A drinking schedule of “1 sip every four minutes” was advocated. Participant core temperature, skin temperature and heart rate were recorded continuously, whilst thermal comfort, response time and cognitive function were assessed at 30-min intervals.

**Results:**

All participants successfully completed their CWT, but only two completed the full 120-mins of their AWT (AWT trial duration ranged from 80 to 120 min). Despite encouragement to drink more, both the rate of consumption (AWT 333 ± 103 v CWT 436 ± 99 ml/h) and total volume of water consumed (AWT 585 ± 233 v CWT 872 ± 198 ml) were less in the AWT (*p* < 0.005). At the 75-min point of the trials, participant core temperatures had increased by 1.26 ± 0.29 in AWT and 0.81 ± 0.30 in CWT. Furthermore, at the point of trial cessation, core temperature in the AWT had increased by 1.69 ± 0.36°C, but only 1.17 ± 0.52°C in the CWT (*p* < 0.05). Participants reported less discomfort and a lower rating of perceived exertion during the CWT. In both trials, response time to the cognitive test decreased as the trials progressed, with no evident difference in response time nor cognitive function between the two trials.

**Discussion:**

The ingestion of cold water was associated with an ability to continue with volitional performance and associated with an ameliorated increase in core temperature as well as providing psychological benefits of cold “refreshment”.

## Introduction

Euhydration and the maintenance of a core temperature within closely defined limits are essential for optimal functioning of the body, whilst an elevated core temperature and dehydration are associated with physical fatigue (1), impaired cognitive function and detriments to both aerobic and anaerobic performance (2–6). Hot environments (>35°C) present a challenge to the thermoregulatory system, particularly during physical activity that increases the rate of metabolic heat production and sweating. A strategy for coping with such environments is the ingestion of cold fluids, which has been shown to be beneficial in endurance activities such as running and cycling (7–11). Additionally, Jay and Morris (10), concluded that the benefits of ingesting cold fluid may be particularly beneficial to those for whom cooling via the evaporation of sweat is impeded. One such group is motor racing drivers who are often required to race for prolonged durations (>90 min), generating metabolic heat due to the work involved, often in hot conditions, with cockpit temperatures exceeding 40°C due to the heat generated by the car as well as the high environmental temperatures of some event locations (12). For this group, these thermoregulatory challenges are compounded by the wearing of compulsory protective clothing and devices, which impair the loss of excess heat by restricting the evaporation of sweat, thus resulting in uncompensable heat stress. This thereby presents participants with an extreme thermoregulatory challenge that can result in increases in core temperature that may exceed 39°C, dehydration of >1% body mass, and thermal discomfort, which may adversely affect physical, cognitive and overall mental performance, as well as elevating health and safety risks (13–15). Hence, in these situations the “heat sink” provided by ingested cold fluid or ice slushies can ameliorate the increase in core temperature and reduce thermal discomfort (2, 8, 15).

A further stimulus for the research described in this manuscript arose from discussions with a former Formula One (F1) driver who had worked professionally with one of the researchers in this study and who recalled how ice cold drinking fluid was placed in the car before the start of the race (before the car was taken onto the grid), but the fluid then being exposed to the heat of the cockpit, rapidly increased in temperature, resulting in it providing “less than optimal” refreshment during the race. At this time of writing in 2024, there is no safety or performance provision for maintaining cold drinking fluids in the racing car during F1 races, many of which take place in extreme hot weather conditions. It was also noted that the volume of fluid available was limited due to concerns about additional weight affecting the performance of the car. Furthermore, whilst some motorsport races allow direct driver cooling systems that may reduce driver heat stress, others, such as F1 do not (16). Therefore, since previous studies investigating the potential benefits of cold fluid ingestion have reported ameliorated increases in core temperature, thermal discomfort and performance detriments, this study extended this work into a sporting scenario in which the protective clothing and device requirements impede thermoregulation, and hence the potential benefits could be even more significant. The aims of this study were therefore to explore whether, in simulated motor racing car cockpit conditions (40°C and 50% humidity), the ingestion of cold fluid compared with ambient temperature fluid affected: (i) thermoregulation, as primarily indicated by any changes in core temperature; (ii) comfort; (iii) cognitive function and; (iv) response time to a cognitive test.

## Materials and methods

### Ethics approval and consent to participate

The study (#20890) was approved by RMIT University Human Research Ethics Committee, and all aspects of the study were undertaken in accordance with the approved procedures, which included the presence of a medical doctor for all trials. All participants provided written informed consent.

### Study participants

Participants were contracted motor car racing drivers known to the researchers and motor car racing drivers from the institution's motor racing club with at least 2-years' experience. Based on the advice of a former F1 driver and one of the researchers who had worked and published in the field of motor racing, the inclusion criteria were chosen to be compatible with the characteristics of those participating in the sport. These included a good level of aerobic fitness, with a measured peak VO_2_ > 45 ml/kg/min during an incremental maximal cycle ergometer test (recording expired air via a Parvomedics metabolic cart, during 3-min stages with 25-Watt increments) and a relatively lean body mass with a sum of four skinfold sites < 55 mm (17, 18). With these also likely to affect prospective participants' capacity to undertake the trials and thermoregulate in hot conditions. Exclusion criteria were contraindications to maximal exercise in hot conditions and blood pressure (SBP > 140 or DBP > 90 mmHg) (19). Participants who passed the screening were familiarized with the climate-controlled chamber, and the assessment tasks and procedures that would be used in the trials.

### General study design

The study used a repeated-measures, cross over design, with participants completing two trials (simulated 120-min races) in a climate-controlled chamber at 40°C and 50% humidity. The variable between the two trials was the temperature of the fluid they ingested, with one trial providing cold water at a temperature of approximately 5°C (CWT), whereas in the other trial the water was exposed to the temperature of the environment and hence was close to ambient (40°C) temperature (AWT). The CWT and AWT trials were undertaken in a balanced random order determined by participants selecting an unmarked envelope from a pack of envelopes prepared by a technician who was not present at the trials. The first trial was undertaken at least two days after the participant's screening, fitness test and familiarization session, and each trial was separated by 7–14 days. At least one researcher always remained in the chamber with the participant and provided instructions, whilst other members of the research team observed the trial through the glass window, monitored the data being collected and manually recorded data on computers located in the adjoining observation area. Verbal communication was maintained between the climate-controlled chamber and observation area via a microphone and speakers.

During the 120-min simulated race, participants were provided with 1 L of drinking water and instructed to ingest 1 sip (∼30 ml) of fluid every 4 min or as close as possible to this time as dictated by their engagement with other tasks during the simulated race. This drinking strategy was adopted in accordance with drinking strategies used by motor racing drivers, as advised by a former F1 driver. The rate of fluid ingestion was monitored throughout the simulated race and the participants encouraged to take larger or smaller sips at each drink, to ingest the 1 L at a consistent rate throughout the trial.

A trial was terminated if the participant's core temperature exceeded 39.5°C in accordance with the study's ethics approval and could also be terminated by the participant at any time, if they felt dizzy, unwell or too uncomfortable. After the participants completed their second trial, they were asked during the recovery time to describe their experiences and provide their perceptions and comments on the two trials.

### Facilities, equipment and instrumentation used for data collection

The motor racing clothing worn during the trials met the Federation Internationale de l'Automobile (FIA) requirements for racing drivers and was as follows: helmet, balaclava, long sleeved top, long johns (close fitting legwear), socks, boots, suit, gloves and neck brace (Hans device—commercially available neck restraint worn to protect the neck from sudden head movements). To standardise moisture content in suit materials across all trials and to thereby eliminate any influence of different moisture content on comfort and participants' next to skin microclimate and their temperature, all suits were kept (conditioned) in the same standardised environmental conditions for a minimum of 12 h prior to each trial at relative humidity 65% ± 2% and temperature 21°C ± 1°C.

Throughout the trials, core temperature was continually assessed using an ingested temperature sensor (CorTemp—T_pill_), which transmitted temperature data to an external receiver. For participants undertaking trials in the morning, the T_pill_ was ingested the night before with food, while for those undertaking trials in the afternoon or evening, the T_pill_ was ingested with breakfast. The timing of ingestion was to ensure adequate descent of the T_pill_ down the gastrointestinal (GI) tract so that the ingestion of water did not transiently interfere with GI temperature measurements. Participants who were unsure whether they were likely to “pass” the pill before the trial commenced were asked to ingest two T_pills_, one the night before and one during the morning of the trial, with the first ingested pill being used to measure core temperature if both were still present at the commencement of the trial, which was the case for all such participants. Skin temperature and microclimate humidity within the participants' racing suits were assessed using 6 temperature loggers (iButton DS1922l, Thermochron) taped to their skin (calf, thigh, 2×chest, lower back and forearm), set at a sampling frequency of once per minute. Ibuttons were also placed in the drinking fluid to record its temperature throughout the trials. After each trial the iButtons were placed in a reader and data downloaded, with data being recorded for the designated time-points. Heart rate was recorded using a Polar heart rate monitor. For the determination of hydration status, the specific gravity of participants' urine samples were analyzed using a refractometer (Atago: Master-URC/NM, cat. No. 2793, Tokyo, Japan).

Perceived exertion was rated using the 6–20 Borg Scale (20), thermal sensation was rated from 1 to 13 and thermal discomfort from 1 to 5 using validated scales (21). Drinking water temperature was rated by the participants on a 5-point scale with descriptors of (1 = Cold, 2 = Cool, 3 = Tepid, 4 = Warm and 5 = Hot).

To assess reaction time and cognitive function, a validated 'Subtle Cognitive Impairment Test' (SCIT) was used (22). Whilst this test had not previously been used with this population, it was selected, as it could be completed within a couple of minutes, unlike other test batteries that have been used in studies investigating thermoregulation but require a longer duration (8–15 min) to complete (23). As this prevented their use in this study, given the required repeated testing and the participants needing to engage in other activities during the trials. In the SCIT, participants were briefly shown a pair of lines and asked to indicate which side was the shortest by pressing either the left or right mouse button. The stimulus was repeatedly presented in a pseudo-random order for exposure durations in the range of 16–176 msec, in 16 msec increments. A total of 22 exposures were used in each SCIT assessment. In the analysis, exposures of 16–64 msec were classified as short exposures and those of 80–176 msec as long exposures. The speed of participant response and the number of errors were recorded for analysis.

### Trials

**Figure 1:**
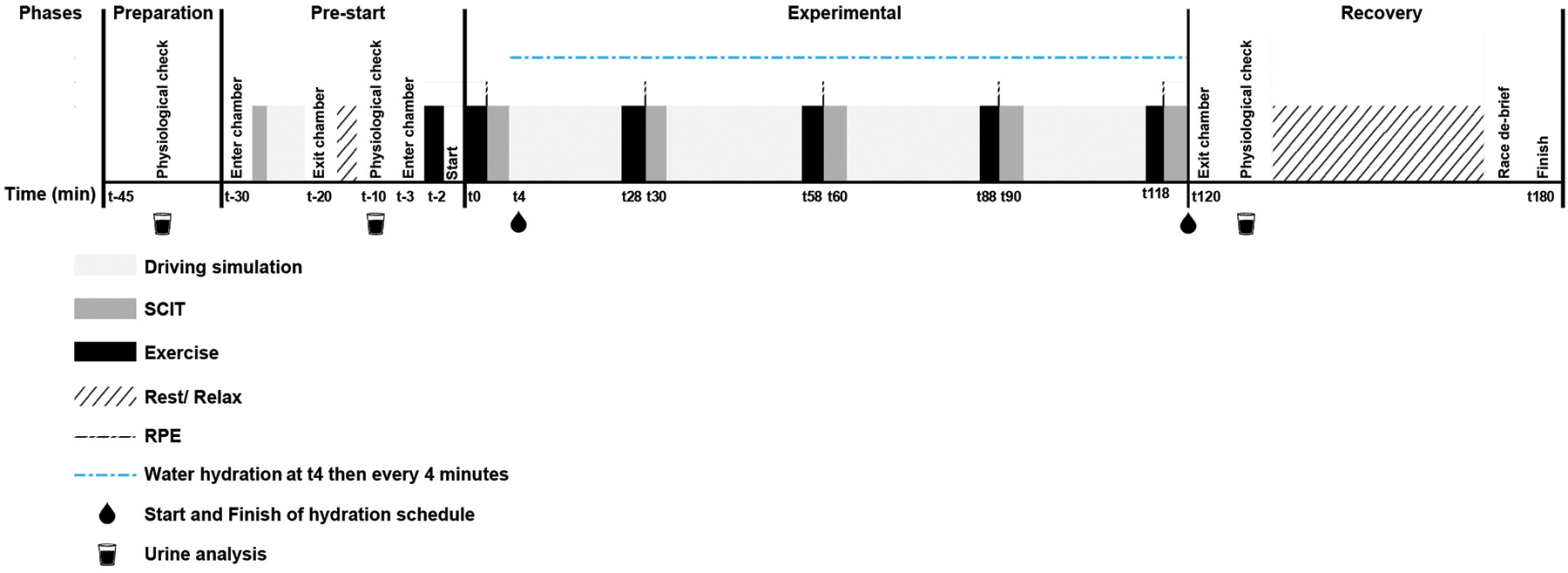
Timeline for each trial.

Each trial comprised of: (i) Preparation time; (ii) a 30-min “pre-start” phase; (iii) a simulated race, scheduled to be of 120-mins duration; and (iv) one hour recovery in an adjoining area at 18–22°C. The schema for the trials is presented in Figure 1. Pilot trials confirmed the validity and feasibility of the trial design in the context of changes to core temperature and reductions in body mass, as these were comparable with published data and data previously collected in the field by a member of the research team during motor racing events (changes in race core temperature being between 2.0 and 3.8°C and reductions in body mass of 2–4 kg) (13, 14, 24).

i. Preparation time: During the preparation time the participants voided their bladders and the specific gravity of their urine sample was measured to determine their hydration status. The timing of this collection was to ascertain their hydration status close to the commencement of the simulated race, since activities during the day may affect this variable, thereby invalidating any collection first thing in the morning. Participants were then weighed in shorts and T shirt. Following this, they donned their motor racing clothing, which had been pre-conditioned overnight and were then re-weighed when fully dressed.
ii. Pre-start phase: At the commencement of the 30-min pre-start phase (t _−30_), the participant entered the climate-controlled chamber in full motor racing clothing ensemble accompanied by two researchers. They completed two sets of the SCIT and checked the driving set-up for the driving simulation game that they would be occupied with during the simulated race. The driving simulation game used was a commercially available computer game that involved simulated motor racing using an interfaced steering wheel and pedals. The purpose of its inclusion was to provide the participants with engagement in a relevant activity during the 120-min trials. No data were collected on their performance in the simulated motor racing game. This took a total of approximately 10 min, after which they exited the climate-controlled-chamber (t _−20_) and returned to the cooler adjoining area (18–21°C), where they were able to remove helmet and balaclava, and unzip and lower the outer driving suit to their waist. They were then able to relax and drink water *ad libitum* before the commencement of the 120-min simulated race. This pre-start phase simulated the pre-race scenario of drivers taking the car out onto the start grid before returning to the pits. Also, at the commencement of this phase a bladder containing 1 L of cooled drinking water (3.5–5.0°C) was placed in the climate-controlled chamber. This is consistent with typical pre-Formula One race drink preparation. However, whilst in the AWTs the “cooled water” was exposed to the 40°C heat of the chamber and thus simulated temperatures changes to the drinking fluid in a “hot racing environment”, in the CWTs the drinks bladder was placed in a refrigerator located within the climate-controlled chamber, which maintained its cold temperature. Ten minutes before the scheduled 'start' of the simulated 120-min race (t _−10_) the participant again voided their bladder, and their urine sample was again analyzed. They were then reweighed in full ensemble.
  Three minutes before the 'start' of the 120-min simulated race (t _−3_) the participant returned to the climate-controlled chamber in full ensemble and at t_−2_ commenced a 2-min cycle ergometry exercise bout at a moderate intensity (60% of the maximum power they attained during their aerobic fitness test). This corresponded with a “warm-up lap” in a race and generated additional metabolic heat, as would be produced by drivers because of the physical exertion involved. This also served to elevate the participants' heart rate before completing the first SCIT test battery. During the final 15 s of the exercise bout the participant indicated their rating of perceived exertion (20), thermal sensation and thermal discomfort (21).
iii. One-hundred and twenty minute simulated race: At t_0_ the participant dismounted from the cycle ergometer and sat at a desk where they completed two sets of the SCIT. They then remained seated and engaged in a motor racing simulation game using a computer with interfaced steering wheel and pedals. The purpose of the driving simulation was to provide mental engagement throughout the trial, but no data were collected from this activity. Throughout the trial they were guided to follow the drinking strategy of 1 sip every 4 min. At t_28_ the two-minute exercise bout was repeated, the purpose of the exercise bouts during the trials was to simulate the generation of metabolic heat due to physical exertion, as would be the case during a motor race, due to the physical demands of driving. This was followed by the reassessment of their perceived exertion, thermo-sensation and thermo-discomfort. They then completed two sets of the SCIT and continued with the driving simulation game. This sequence was repeated every 30 min at time points: t_58_, t_88_ and t_118_.
iv. Recovery: Upon completion of the final SCIT test battery at ∼t_125_, the participant left the climate-controlled chamber and was weighed in full ensemble. They then removed their driving ensemble, changed into a dry t-shirt and shorts and were weighed again. If they were able, they then voided their bladder. The collected urine volume was included in the calculations and its specific gravity assessed. Post-trial, the amount of drinking fluid remaining was recorded and subtracted from the original 1-L volume to determine the volume ingested during the trial. All ensembles were reweighed to determine the amount of sweat absorbed into the clothing. The participant then remained in the adjacent observation room, which was set at a cool temperature of 18–20°C for post-trial recovery. Here they were provided with electrolyte fluids and/or water *ad libitum*. Participants showered when ready and remained at the facility for 1 h until deemed ready to leave, as confirmed through a post-test debriefing and questionnaire.

### Data analysis

Whilst data collection was continuous for some variables, for the purposes of data analysis, data were sampled at the specified timepoints in the trials. Since not all participants completed the entire 120-mins of the AWT, the data for the CWT and AWT trials are described and compared using: (i) the absolute values at the end of each trial regardless of trial duration (120 min for all CWT but ≤120 min for AWT); (ii) the amount of change of a variable between the start of the trial and trial cessation, calculated from “final value—initial value” (120 min for all CWT but ≤120 min for AWT); (iii) the rate of change (“final value—initial value”/trial duration) between the start of the trial and trial cessation (120 min for all CWT but ≤120 min for AWT); (iv) using the absolute values for the last data point available for each variable prior to the t_80_ time point, as this was reached by all participants in the AWT trials; (v) calculating the rate of change for each variable between the start of the trial and last data point available for each variable prior to the t_80_ point (‘value at or immediately prior to t_80_—initial value at or immediately after t _0_/time between recording the two values).

Data were checked for normality (Kurtosis and Skewness), with statistical comparisons being made using paired *t*-tests with *α* = 0.05 and descriptive data presented as means ± SD. When technical difficulties resulted in missing data points, these participants are excluded from the analysis of that variable, and this is reflected in the reported number of participants in the results.

## Results

### Experimental conditions

The temperature and humidity of the climate-controlled chamber during the AWT were 40.4 ± 1.4°C and 52.2 ± 3.0%, and for the CWT, 40.1 ± 0.9°C and 52.3 ± 2.6%. At the commencement of the 120-min race simulation (t_0_), the temperature of the drinking water in the AWT had already increased to 28.9 ± 2.4°C, while in the CWT it was 4.0 ± 1.2°C. As the trial progressed the temperature of the drinking water in the AWT increased to 37.7 ± 2.0°C at t_80_, whereas in the CWT it had only increased to 6.3 ± 1.8°C by the end of the trial at t_120_.

### Effect of trial conditions on drop-out and trial cessation time

Eight volunteers commenced the trials (Table 1), with four undertaking the CWT first and four undertaking the AWT first. All participants successfully completed the full 120-mins of their CWT but only 2 completed the full 120-mins of their AWT (Tables 2, 3). The duration of the AWT trials were 80–120 min, with the one participant who reached 120 min exiting the climate-controlled chamber immediately after the final exercise bout before completing the final SCIT. Reasons for terminating the AWT before the scheduled end of the 120-min duration were due to the participants indicating that they felt dizzy and/or unable to concentrate and/or “had had enough”.

**Table 1:** Participant demographics (*n* = 8) (mean ± SD).

Age (yr)	22 ± 4
Height (m)	1.81 ± 0.06
Weight (kg)	75.8 ± 9.9
Peak VO_2_ (ml/kg/min)	52.0 ± 7.3
Sum of 4 skinfolds (mm)	38.3 ± 9.5
Systolic blood pressure (mmHg)	125 ± 10
Diastolic blood pressure (mmHg)	68 ± 7
Cycling watts used in trials (60% VO_2 peak_)	150 ± 17

Despite the researchers encouraging the participants to drink more (take larger sips of fluid at each drink) during the AWT when participants fell behind the scheduled rate of ingestion, a greater volume of fluid was ingested in the CWT both in absolute volume and volume drunk per hour.

### Physiological variables

**Figure 2:**
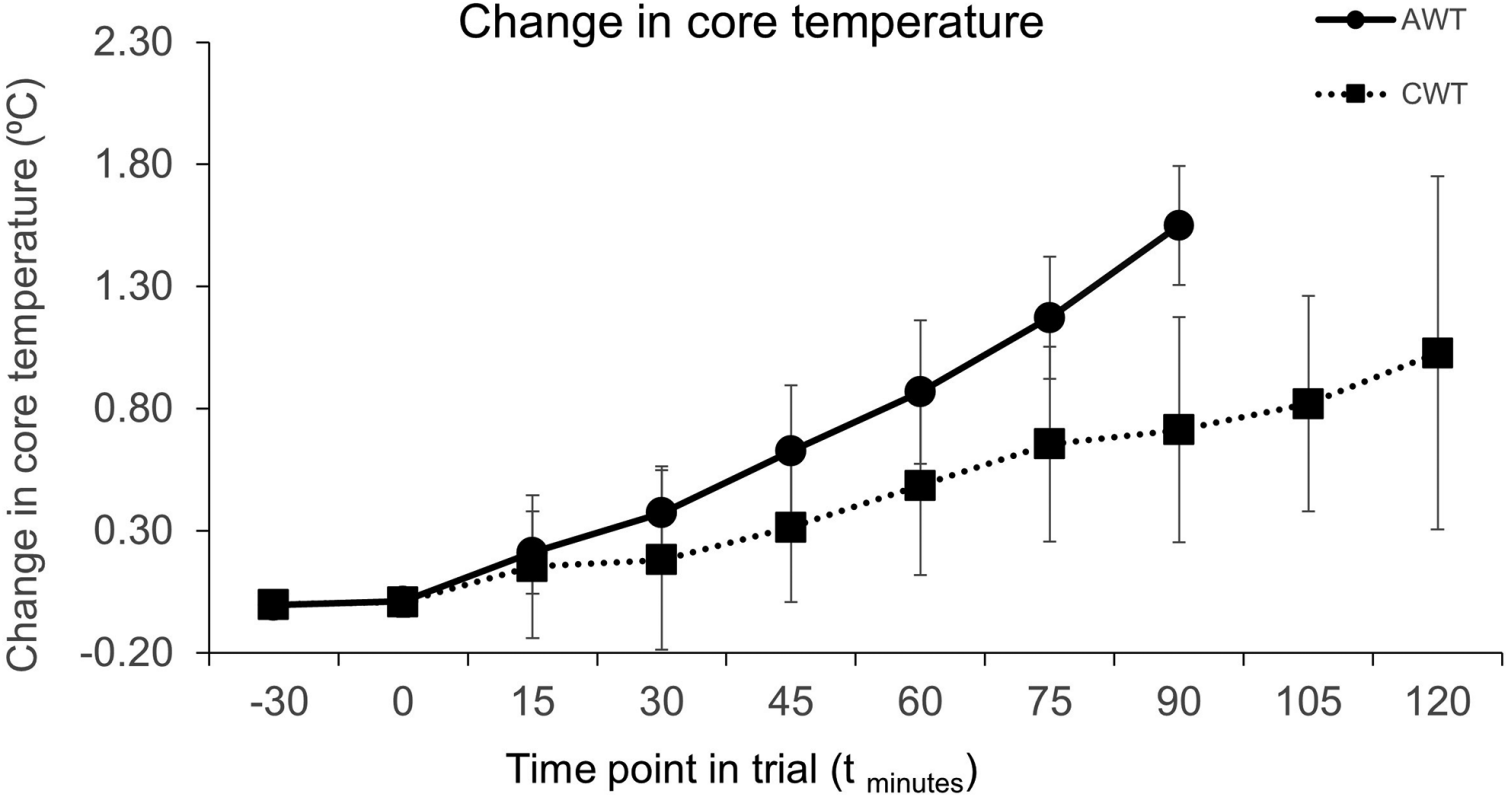
Change in core temperature during trials. Commencement of race simulation is at t_0_. CWT – Cold Water Trial, AWT, ambient WATER trial. Error bars are SD for change in temperature from t_−30_ (*n* = 6). AWT data are not shown after t_90_ as only two participants reached t_120_ in their AWT trial.

During the trials the core temperature of the participants increased steadily in both trials (Figure 2), with the increase in core temperature being greater in the AWT both in absolute values and relative rate of increase (*p* < 0.05). In both trials, skin temperature rapidly increased during the first 15 min by approximately 4.5°C and then remained constant for the remaining duration of the trials, with no statistically significant difference between trials.

**Table 2:** Physiological data reported as absolute change between start of simulated race (t_0_) and end of trial, regardless of simulated race duration, and as rate of change.

	*n*	AWT	CWT	Difference AWT and CWT	*p*
Volume drunk (ml)	8	585 ± 233	872 ± 198	288 ± 192	0.003*
Volume drunk ml/hr	8	333 ± 103	436 ± 99	103 ± 74	0.005*
Gross body mass loss (primarily sweat lost during trial) (g)	8	1,346 ± 429	1,475 ± 395	129 ± 306	0.274
Gross body mass loss (primarily sweating rate during trial) (g/hr)	8	781 ± 222	737 ± 198	44 ± 217	0.586
Net change in body mass (kg)	8	0.85 ± 0.33	0.70 ± 0.40	0.15 ± 0.32	0.227
Net change in body mass (kg/h)	8	0.50 ± 0.21	0.35 ± 0.20	0.15 ± 0.22	0.097
Core temperature at cessation of trial (⁰C) t = 120 min for CWT but variable for AWT, t ≤120 min	6	38.66 ± 1.16	38.06 ± 1.26	0.60 ± 1.15	0.259
Change in core temperature during trial (⁰C) t_120_ - t_0_ for CWT but variable for AWT t_≤120_ - t_0_	6	1.69 ± 0.36	1.17 ± 0.52	0.52 ± 0.41	0.026*
Core temperature at t = 75 min (⁰C)	6	38.23 ± 1.17	37.70 ± 0.93	0.53 ± 0.97	0.238
Change in core temperature from start to 75 min t_75_ - t_0_ (⁰C)	6	1.26 ± 0.29	0.81 ± 0.30	0.45 ± 0.18	0.002*
Skin temperature at cessation of trial (⁰C) (t = 120 min for CWT but variable for AWT (t ≤ 120 min)	7	37.36 ± 0.27	37.64 ± 0.25	−0.28 ± 0.41	0.120
Change in Skin temperature during trial (t_=<120_ - t_−5_ min) (⁰C)	7	4.61 ± 0.70	4.55 ± 0.52	0.06 ± 0.78	0.845
Skin temperature at t_75_ (⁰C)	7	37.23 ± 0.21	37.47 ± 0.20	−0.24 ± 0.33	0.101
Change in skin temperature from start to 75 min t_75_ – t_−5_ (⁰C)	7	4.47 ± 0.76	4.38 ± 0.50	0.10 ± 0.72	0.731
Exercise heart rate in final exercise bout (bpm) t_118_ min for CWT but variable for AWT t _≤118_	8	180 ± 11	188 ± 11	−8 ± 11	0.107
Difference in exercise heart rate between first and last exercise bout (bpm) t_118_ – t_0_ for CWT but variable for AWT t _≤118_ -t_0_	8	37 ± 10	38 ± 7	1 ± 10	0.877
Exercise heart rate at 60 min t_60_ (bpm)	8	171 ± 6	179 ± 12	−7 ± 10	0.100
Difference exercise heart rate between first bout and bout at 60 min t_60_ – t_0_ (bpm)	8	27 ± 11	28 ± 7	1 ± 7	0.734
Non exercise heart rate at cessation of trial (bpm) t_118_ min for CWT but variable for AWT t_≤118 min_	8	131 ± 14	133 ± 12	2 ± 5	0.338
Change in non-exercise heart rate during trial (bpm)	8	39 ± 18	41 ± 14	2 ± 14	0.703
Non-exercise heart rate at t_80_ min (bpm)	8	126 ± 15	120 ± 11	6 ± 12	0.181
Change in non-exercise heart rate from start to 80 min (bpm) t_80 –_ t_−2_ min for CWT but variable for AWT t_≤80 min_ – t_−2_	8	27 ± 14	22 ± 14	7 ± 12	0.185
Urine specific gravity pre-trial	8	1.019 ± 0.008	1.012 ± 0.006	0.006 ± 0.007	0.076
Urine specific gravity post-trial	8	1.021 ± 0.006	1.022 ± 0.007	0.001 ± 0.011	0.804
Change in urine specific gravity	8	0.002 ± 0.007	0.008 ± 0.008	0.006 ± 0.012	0.242
Change in urine specific gravity/hr	8	0.001 ± 0.004	0.004 ± 0.003	0.003 ± 0.006	0.261

In CWT duration of the simulated race was 120 min for all participants but ranged from 80 to 120 min in AWT, as some participants terminated their trial before the scheduled 120 min duration.

* *P* < 0.05.

There was no statistically significant difference in the loss in body mass (primarily sweat loss) between the two trials, either in absolute loss or rate of loss. Similarly, there was no difference in the specific gravity of the urine samples between conditions pre-trial, post-trial or rate of change.

The participants' heart rates recorded at the end of each exercise bout increased as the trials progressed, indicating increasing physiological stress, despite the exercise work rate for each participant being the same for each exercise bout within their trials. However, there was no statistically significant difference in “exercise heart rate” at any timepoint between trials. Additionally, the increasing physiological stress experienced as the trial progressed was also evident in the increasing heart rate recorded between the exercise bouts (non-exercise heart rate) whilst the participants were engaged in the simulated driving activity, but again there was no difference between trials at any timepoint.

### Thermal sensation/comfort, perception of water temperature and qualitative data

The participants' perceptions of the drinking water in the AWTs trial commenced as tepid and progressed to warm or hot, whereas in the CWTs the water was deemed as “cold” throughout (AWT v CWT, *p* < 0.001). The participants’ thermal sensation rating and discomfort increased as each trial progressed and there was a greater rating of thermal discomfort at the end of the AWT compared to the CWT. Aligned with this, the rating of perceived exertion (RPE) during the cycling exercise at t_60_ was higher in the AWT (*p* < 0.05), despite no statistically significant differences between trials in heart rate during the exercise bout at this timepoint.

**Table 3:** Comparison of psychological data between ambient water trials (AWT) and cold water trials (CWT).

	*n*	AWT	CWT	Difference AWT - CWT	*p*
Exercise RPE at cessation of trial t_120_ min for CWT but variable for AWT t _≤120_	8	16.5 ± 2.4	16.4 ± 1.8	0.1 ± 2.1	0.871
Change in RPE during trial	8	5.3 ± 1.6	5.1 ± 2.4	−0.2 ± 1.6	0.750
Exercise RPE at t_60 min_	8	13.9 ± 2.1	12.9 ± 1.6	1.2 ± 1.2	0.033*
Thermal discomfort at cessation of trial (1–5) t = 120 min for CWT but variable for AWT, t ≤ 120 min	8	4.5 ± 0.7	4.1 ± 0.5	0.4 ± 0.5	0.041*
Change in thermal discomfort between start and cessation of trial (1–5) t_120_ – t_0_ for CWT but variable for AWT, t _≤120_ – t_0_	8	2.3 ± 0.8	2.1 ± 0.6	0.1 ± 0.8	0.684
Thermal discomfort at 60 min t_60_ (1–5)	8	3.8 ± 0.7	3.5 ± 0.5	0.3 ± 0.7	0.351
Change in thermal discomfort between start of trial and 60 min t_60_ – t_0_ (1–5)	8	1.5 ± 0.5	1.6 ± 0.5	−0.1 ± 0.5	0.731
Thermal sensation at cessation of trial (1- 13) t = 120 min for CWT but variable for AWT, t ≤ 120 min	8	11.3 ± 1.0	11.3 ± 0.7	0.0 ± 0.7	1.000
Thermal sensation at 60 min (1–13)	8	10.5 ± 0.8	10.2 ± 0.5	0.3 ± 0.5	0.170

* *p* < 0.05.

During the post-trial debrief, participants unanimously reported a preference for the cold water, which was perceived as refreshing, and one reported that “*things were clearer after the cold water*” and they were better able to concentrate. Whereas the ambient water was not favored by any participants and some also specifically stated that they did not want to drink the warm/hot water in the AWT, a point that was evident in the lower rates of drinking despite encouragement from the researchers to keep to the prescribed rate.

### Cognitive function

**Figure 3:**
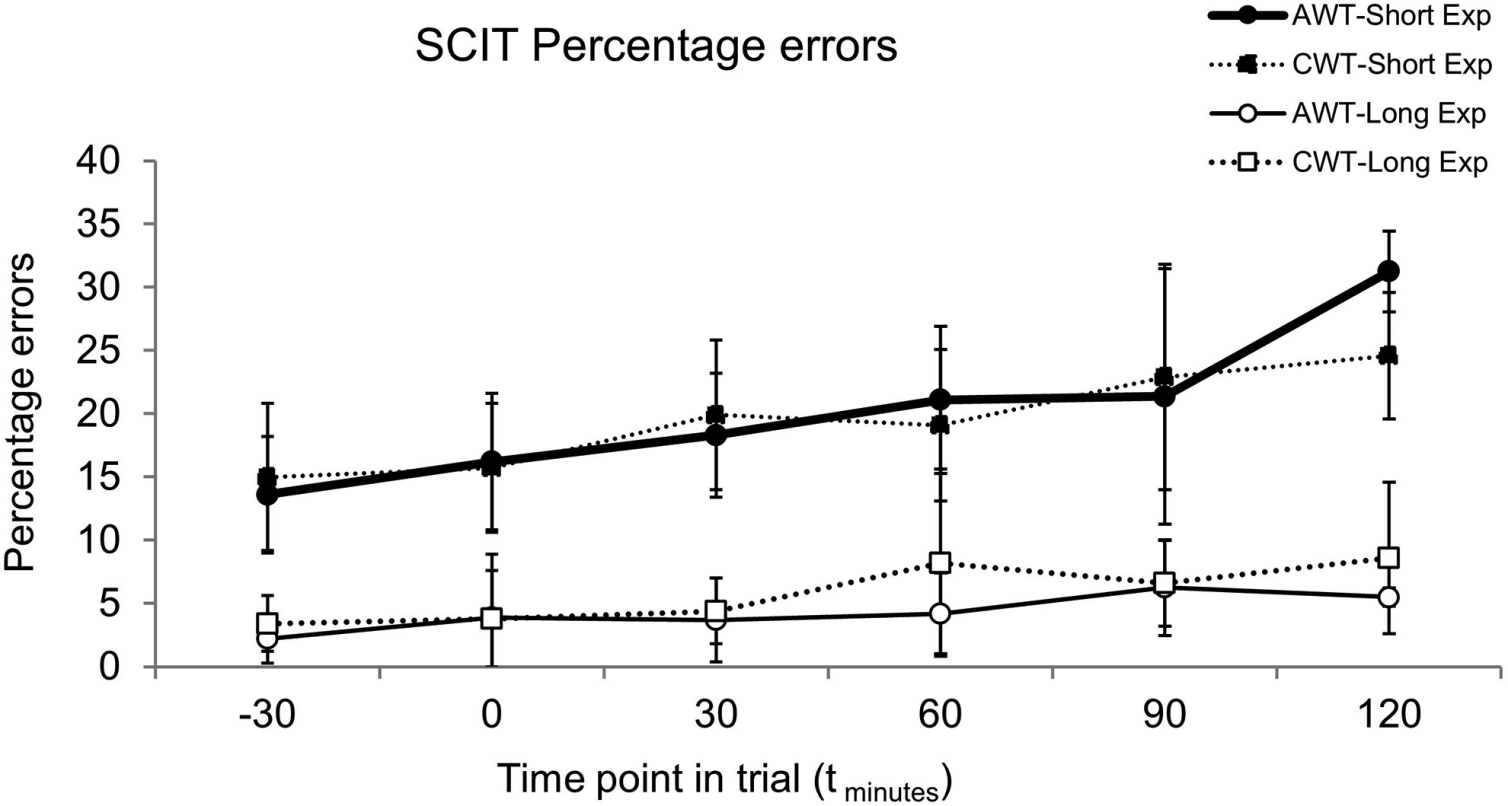
SCIT percentage errors at time points during trials for long exposure (long Exp) and short exposure (short Exp) stimuli, error bars are SD. CWT, cold water trial, AWT, ambient water trial. Data for each timepoint are only shown for participants who reached that timepoint in both their AWT and CWT, thus for timepoints t_−30_ to t_60_ *n* = 8, for timepoint t_90_ *n* = 7, and at t_120_ *n* = 2. Simulated race commenced at t_0_.

In the SCIT, the participants made a greater percentage of errors in decision making for the short exposure stimuli (visible for 16–64 ms) compared with the longer exposure stimuli (visible for 80–128 ms) (Figure 3). This is likely to be due to the short exposure presentation times of 16–64 msec being, processed without conscious awareness, whilst the longer exposure presentation times of 80–176 msec incorporate a conscious attention component (22). Additionally, the results showed an increase in the percentage of errors as the trial progressed. However, despite participants indicating in their post-trial comments that they were finding it harder to concentrate in the AWT, this was not reflected in the SCIT data, which did not show any statistically significant differences in error rates between trials. Indeed, the magnitude of difference errors between trials was less than 2%, except at t_120_, at which point there were only two participants remaining in the AWT when it increased to 6%, but given the small number of participants at t_120_, this should be viewed with caution.

**Figure 4:**
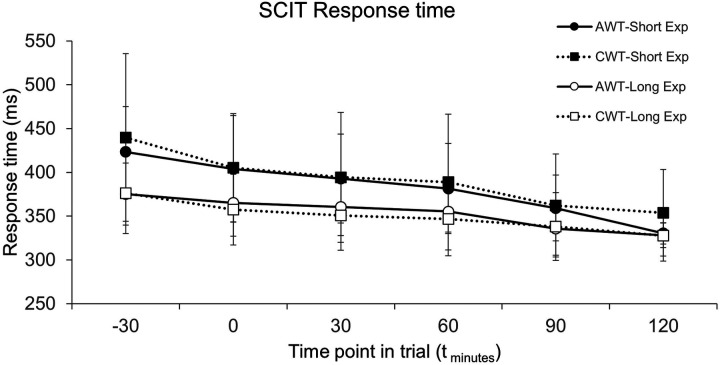
SCIT response time at points during trials for long exposure (long Exp) and short exposure (short Exp) stimuli, error bars are SD. CWT, cold water trial; AWT, ambient water trial. Data for each timepoint are only shown for participants who reached that timepoint in both their AWT and CWT, thus for timepoints t_−30_ to t_60_ *n* = 8, for timepoint t_90_ *n* = 7, and at t_120_ *n* = 2. Simulated race commenced at t_0_.

As expected, response times to the long exposure stimuli were faster than the short exposure stimuli but did not differ between trials. Furthermore, in both trials the response times to both the short and long exposure stimuli became significantly faster as the trials progressed (Figure 4).

## Discussion

The simulated race conditions generated, and participant responses recorded in this study were similar to the race data reported by Brearley and Finn (13), which included: post-race driver core temperatures of 39.0°C ± 0.4°C; driver heart rates of >170 bpm; sweat rates of 1.06 ± 0.12 L/h; thermal sensation as hot (10.3 ± 0.9), thermal discomfort as uncomfortable (3.1 ± 1.0), and perceived exertion as very hard to very, very hard. This therefore suggests that the simulated race conditions and design used in the current study provided an appropriate level of physiological stress and thereby indicates ecological validity in the trials for comparing the effects of cold-water vs. ambient temperature water ingestion.

One of the most striking aspects of the trials was six of the eight participants terminating the simulated race in the AWT before the scheduled 120-min end point. This was due to their perceived discomfort and occurred despite some having already successfully completed the full 120-mins in the CWT and hence the failure to complete the full 120 min cannot be ascribed to a lack of familiarity with the trial protocol or demands. One of these participant's core temperature reached the predetermined value for trial termination (>39.5°C), and their AWT trial would have been terminated by the researchers at this point, if the participant had not terminated it themselves. The reasons for the reduced completion rates in the AWT are likely to be due to a combination of the greater increase in core temperature, which was ameliorated by the ingested cold water in the CWT, and also the psychological impact of the cold-water ingestion, which the participants reported as being refreshing, whereas the ambient water was not. The impact of the cold fluid in providing a “heat sink” that ameliorated increases in core temperature and reduced thermal discomfort was similar to that reported in a study with cyclists (7, 8). Furthermore, the difference in palatability was commented on in the participants' de-briefing and was reflected in the slower rate of drinking in AWTs: which was below that of the target of 500 ml/hr, despite the researchers encouraging participants to take larger sips at each drink, to maintain the rate of fluid intake.

Compared to the AWTs, thermal discomfort was less at the end of the CWTs and the participants' exercise RPE was lower in the CWTs 60-mins into the trial, both of which may contribute to the reasons why some participants terminated their AWT before the scheduled maximum of 120-mins race simulation, but were able to complete the CWT. These differences in core temperature, thermal discomfort and RPE, reflect the findings of Burdon et al. (7, 8), with cyclists and Stevens et al. (9), in their review, who report the importance of psychophysiological factors that contribute towards performance, and a potential role for cold sensors in the mouth and gastrointestinal tract. Hence both the physiological impact of the cold water in ameliorating the increase in core temperature by acting as a “heat sink” and its psychological impact may be important.

The lack of a significant effect of cold fluid ingestion on skin temperatures between the CWT and AWT, differs from some studies that have reported a lower skin temperature following the ingestion of cold fluid during cycling, albeit that in these other studies the participants were not required to wear the thermoregulatory impeding protective clothing (25, 26), which is a unique feature of the study reported here.

The faster response times recorded as the trials progressed concurs with previous studies that have found a reduction in response time with increased core temperature (27, 28). Additionally, the progressive increase in percentage errors concurs with some previous research on the impact of elevated core temperatures (5, 27) but differs from others that have found no effect (29). In our study we did not find evidence for any benefits of cold-water ingestion on cognitive function, which concurs with some studies that have found no statistically significant effect of cooling strategies on cognitive performance during tasks with an elevated core temperature (28) but differs from others that propose some potential benefit (5). The reasons for these inconsistencies could be due to the participants and/or choice of cognitive function test, since many studies tend to use a longer battery of cognitive function tests than that used in the current study. Also, the trial duration may be a factor in these inconsistencies, since Mollica (30) reported no effect in short duration simulated motor racing. Also, inconsistencies in research findings may arise due to the magnitude of increase in core temperature, as Schmidt et al. (5) suggest a threshold of 38.5°C for “hyperthermia-induced negative cognitive performance”.

Furthermore, whilst the CWT clearly produced benefits in ameliorating an increase in core temperature and improving participant comfort, it is unknown whether these differences can be definitively attributed to the temperature and/or volume of the ingested fluid. Since, whilst the trials provided drinking water of different temperatures, the voluntary nature of the participants’ ingestion of the fluid resulted in different volumes being ingested. This being despite the researchers encouraging the participants to keep to the prescribed rate of ingestion. However, given that the temperature of the water ingested in the AWT was close to the participants' core temperature, the authors would suggest that the temperature of the ingested fluid is likely to be the key factor. All of which suggest that further research is required into hyperthermia, cooling strategies, physiological changes and cognitive performance.

## Conclusions

The findings of this study clearly demonstrate that cold water ingestion can provide a heat sink that ameliorates the potential increases in core temperature, and also has an important effect on perceived thermal stress and comfort. Furthermore, the cold fluid was deemed by the participants to be more palatable and thereby encouraged greater fluid intake, which is likely to reduce the magnitude of dehydration in such circumstances. These combined effects are likely to benefit prolonged performance in hot, humid conditions, such as motor racing, which was simulated in this study. These benefits were associated with persisting with the simulated activity beyond the time point at which participants withdrew from the trials in which they were ingesting ambient temperature fluid. However, based on the available data, no differences were found in response time nor cognitive function between the two trials. The potential thermoregulatory and thermal comfort benefits may also relate to other professions that work in hot environments with protective clothing, such as firefighters, since whilst the importance of the provision of fluids is well established in these contexts, being able to provide these fluids at cold rather than ambient temperatures may be advantageous.

### Limitations

Whilst endeavoring to simulate F1 motor racing conditions, the authors are aware of the limitations in terms of ecological validity. These include the participants completing the trial in a climate-controlled laboratory setting and not in real-world motor races. In which, the physical demands, motivation, focus, concentration and skills would be different. The impact of which is likely to distract the drivers from issues such as “comfort”. And related to this, in real-races, drivers are very unlikely to terminate their drive on the basis of perceive comfort and/or ability to continue, which was the case for several drivers in the AWT. Also, it is also worth noting that given the ethics approval requirements for this study, the maximum core temp was set at 39.5°C, whereas in motor races core temperature can exceed 40°C.

Furthermore, the assessment of cognitive function was a general test and not specific to the cognition needed whilst racing and the execution of driving skills. Additionally, for ethical, safety reasons, as well as the drivers' perceived comfort, most of the drivers terminated their ambient water trial before the completion of the full 120-mins, which is not a normal occurrence in motor racing, despite the aforementioned conditions and ingestion of ambient temperature fluid.

### Impact and application to the field

Despite the extraordinary technological advances made in the global motor sport phenomenon of Formula One racing, drivers accessing cold drinking fluid during a race is yet to be achieved. Despite the aforementioned limitations of the study, the findings suggest that the physiological and psychological benefits of ingesting cold-fluid warrant prioritizing technology that can maintain cold fluids for prolonged periods, in a sport characterized by weight restriction challenges. Furthermore, these benefits may also impact other open wheel motor racing categories, and professions that work in hot environments with protective clothing, such as emergency services and military.

## Data Availability

The raw data supporting the conclusions of this article will be made available by the authors, without undue reservation.
